# Magnetic field dependence of singlet oxygen generation by nanoporous silicon

**DOI:** 10.1186/1556-276X-9-342

**Published:** 2014-07-09

**Authors:** Jamaree Amonkosolpan, Gazi N Aliev, Daniel Wolverson, Paul A Snow, James John Davies

**Affiliations:** 1 Department of Physics, University of Bath, Claverton Down, BA2 7AY Bath, UK

**Keywords:** Singlet oxygen, Photoluminescence, Energy transfer, Porous silicon

## Abstract

Energy transfer from photoexcited excitons localized in silicon nanoparticles to adsorbed oxygen molecules excites them to the reactive singlet spin state. This process has been studied experimentally as a function of nanoparticle size and applied external magnetic field as a test of the accepted understanding of this process in terms of the exchange coupling between the nano-Si exciton and the adsorbed O_2_ molecules.

## Background

Since the discovery that photoexcited silicon nanoparticles can act as energy donors to molecular oxygen acceptors and can thereby excite oxygen to a highly reactive singlet state [1-3], there has been much work on the potential exploitation of this process. Applications that have been demonstrated range from photodynamic cancer therapy [4,5] to optically activated reactors in chemical engineering [6].

In early work, it was demonstrated that the efficiency of the energy transfer process is sensitive to an externally applied magnetic field [2] (the energy transfer efficiency may be monitored by its quenching of the nano-Si residual photoluminescence), and this provided key evidence for the understanding of the process as a result of exchange coupling between an exciton confined within a silicon nanoparticle and an adsorbed oxygen molecule (the Dexter exchange mechanism). The applied magnetic field *B* lifts the spin degeneracy of both the exciton and oxygen spin manifolds; both oxygen molecules and silicon excitons will then relax predominantly into their lowest energy spin states at temperatures *T* for which *gμ*_*B*_*B* ≥ *kT* where *g* = 2.0 is the gyromagnetic ratio and *μ*_*B*_ is the Bohr magneton. The energy transfer process between these lowest energy spin states has a low probability due to angular momentum selection rules, so that the effect of the magnetic field at low temperatures is to suppress the energy transfer from the exciton to the molecular oxygen. As a result, the silicon photoluminescence intensity is restored towards the intensity observed when oxygen is not present.

Although earlier investigations proposed this model [2], the response to a magnetic field has not been investigated or modelled quantitatively in terms of the dynamics of the energy transfer and other excitation and relaxation processes. Furthermore, the dependence of the efficiency of the process on oxygen concentration has never been investigated. Here, we show results of experimental investigations at lower oxygen concentrations than used previously, and we set out a preliminary model which makes some simplifying assumptions but which has the features required to describe our experimental data. This model is a starting point for a full theoretical description of the energy transfer phenomenon and can be expanded to model the energy transfer process as a function of, for example, nanoparticle size. Even at the present level of approximation, the modelling turns out to be a fairly complicated task requiring a large set of input parameters, though many of these are available in the literature; some we use have been estimated as part of the present work.

## Methods

The samples were produced in the form of porous silicon layers (thickness of approximately 8 *μ*m) on bulk crystalline substrates by conventional electrochemical etching from wafers consisting typically of *p*-type boron-doped CZ <100> silicon with resistivities of 1 to 25 *Ω* cm. Room temperature anodization was performed in a 1:1 solution of 49% aqueous HF and hydrous ethanol; the porosity *p* was varied by variation of the current (10 to 40 mA/cm^2^) and was determined by fitting of the Fabry-Pérot interference fringes in a broad-band optical reflectance measurement [7] to be typically *p* = 63% to 70%. The etched layers were left attached to the substrates for better mechanical strength and were glued to a copper cold finger with heater and thermometer resistors attached. The samples were held either in a continuous-flow cryostat (base temperature of approximately 10 K) or a superconducting magnet in superfluid helium (base temperature of approximately 1.5 K). The magnetic field was varied up to 6 T and was oriented either parallel or perpendicular to the sample normal. The orientation of the field plays no role in the following experiments, in which the optical polarisation of the photoluminescence (PL) emission was not analysed. The effects we discuss here depend only on the magnitude of the induced Zeeman splittings in the exciton and oxygen triplet states (polarisation-dependent studies are under way at present). In both cryostats, the cold finger could be raised to the top of the cryostat to expose the cold sample briefly to oxygen gas and it could be heated whilst in vacuum to desorb oyxgen. PL was excited by a continuous wave solid state diode laser (wavelength approximately 450 nm, power approximately 5 mW at the sample, with a weakly focused laser spot, size a few hundred microns) and detected with an intensified CCD camera and compact single-grating spectrometer.

## Results and discussion

**Figure 1:**
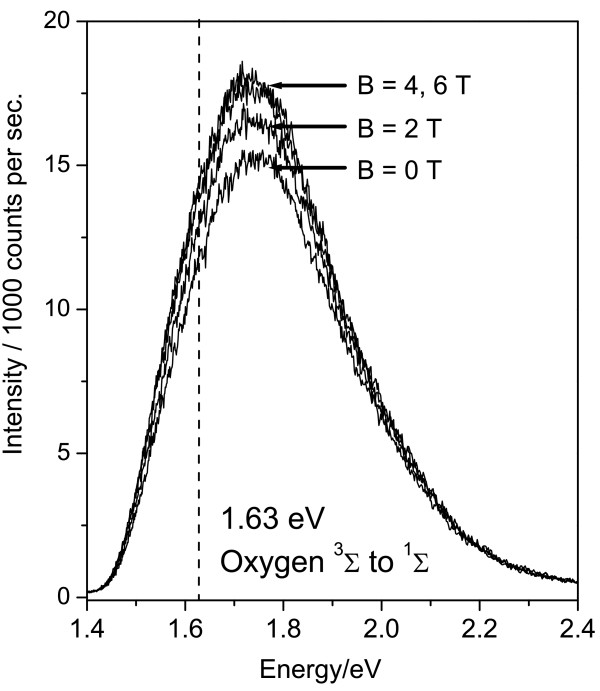
**Photoluminescence of porous silicon containing a low concentration of molecular oxygen.** Photoluminescence (PL) spectra of a porous silicon sample exposed to a small quantity of oxygen gas are shown for magnetic fields of 0 to 6 T. The sample was held in superfluid helium at 1.5 K, and the PL was excited with 450-nm (2.76 eV) continuous wave excitation. The vertical dashed line indicates the threshold energy, above which photoexcited excitons in the silicon nanoparticles have sufficient energy to excite the adsorbed oxygen from its triplet ^3^Σ to its singlet ^1^Σ state.

**Figure 2:**
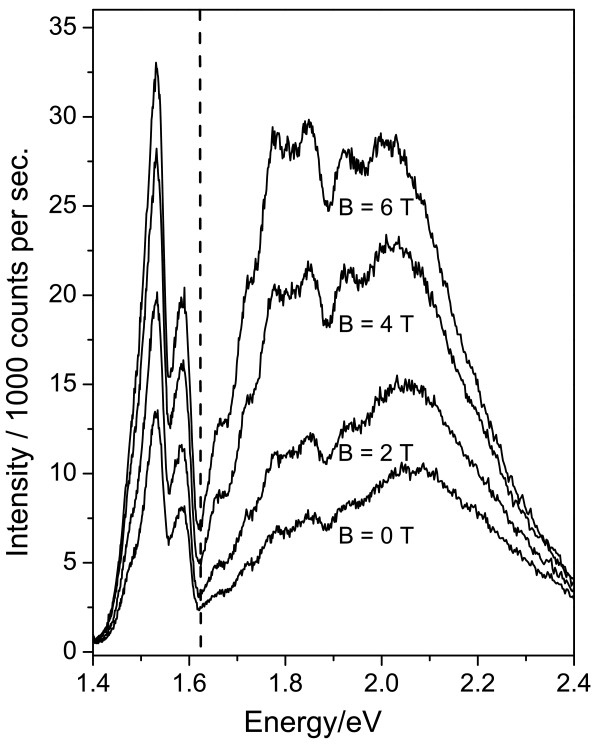
**Photoluminescence of porous silicon containing a high concentration of molecular oxygen.** Photoluminescence (PL) spectra of a porous silicon sample exposed to a larger quantity of oxygen gas than in Figure 1 are shown for magnetic fields of 0 to 6 T. As in Figure 1, the sample was held in superfluid helium at 1.5 K, and the PL was excited with 450-nm (2.76 eV) continuous wave excitation. The vertical dashed line again indicates the threshold energy for energy transfer, at which the quenching of the PL is particularly efficient. Other structures arise from energy transfer processes in which phonons participate.

Four typical PL spectra at 1.5 K for a porous silicon sample exposed to a low oxygen concentration are shown in Figure 1 (spectra were recorded at 0.5-T intervals, but for clarity, we omit the spectra at intermediate fields). The broad luminescence band corresponding to a wide distribution of silicon nanoparticle (NP) sizes is observed [8-10]; this band is similar in shape to that obtained in the absence of oxygen but is lower in intensity. The overall intensity of the PL band increases by about 20% as the applied magnetic field is increased to around 4 T and then ceases to increase further. This behaviour differs quite markedly from the first reported experiments using a magnetic field, where the oxygen concentration was high enough that PL above the threshold energy of 1.63 eV for singlet oxygen production was still completely suppressed even at fields as high as 10 T and the field-induced recovery of the PL intensity was only observed *below* 1.63 eV [2].Figure 2 shows the PL spectra obtained at higher oxygen concentrations (Figure 2) in a second piece of the porous silicon sample used to obtain the results of Figure 1. It is not possible to measure quantitatively the oxygen concentration adsorbed on the silicon NPs, but the much stronger quenching of the PL gives a clear indication that the concentration is higher than in the case of Figure 1.

There are two notable features: Firstly, the strongest quenching of the PL occurs precisely for NPs having an exciton energy equal to the oxygen ^3^Σ to ^1^Σ transition energy of 1.63 eV. Secondly, the spectra show a large number of other sharp downward-pointing peaks or dips which originate from the enhanced energy transfer to oxygen for NPs whose exciton energies differ from 1.63 eV by energies corresponding to one or more momentum- and energy-conserving phonons (located at *K* and Γ points of the silicon phonon dispersion, respectively). These phonon effects have been discussed elsewhere, where details of the relevant phonon energies are given [3]. Two prominent dips of this type can be seen near 1.9 and 2.0 eV; these are also related to energy transfer to oxygen but will be discussed in future work; here, we shall model only the energy transfer process without phonon participation.Figure 2 demonstrates that significant PL is again observed above the threshold for energy transfer to oxygen, even at this higher oxygen concentration. Furthermore, the PL both above and below this threshold shows a much stronger recovery of intensity as the magnetic field is increased, by factor of about 3 times, and unlike the case of Figure 1, the recovery of the PL has not saturated up to a magnetic field of 6 T.

**Figure 3:**
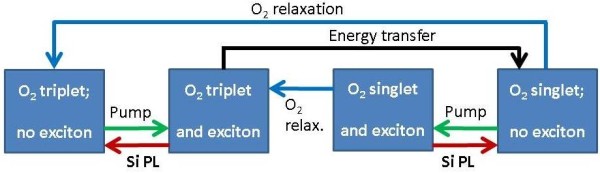
**Schematic overview of energy transfer from photoexcited excitons in silicon nanoparticles to absorbed oxygen molecules.** Optical excitation (green arrows, ‘pump’) generates excitons confined in silicon nanoparticles that can recombine to emit photoluminescence (red arrows, ‘PL’) or can transfer energy to those absorbed oxygen molecules that are in the triplet ground state (black arrow, ‘energy transfer’). Excited oxygen molecules in the singlet state can return to their ground state (blue arrows, ‘relaxation’) via emission of luminescence and/or non-radiative relaxation processes.

The differences between Figures 1 and 2 point to an interplay between the rates for the physical processes (light absorption, radiative recombination, spin relaxation, and energy transfer) that control the shape of the PL spectrum. These processes are indicated schematically in Figure 3, which serves as a guide to the rate equation model we develop below. Figure 3 summarises the situation of NPs with oxygen present, for which there are four possible states (represented by the four boxes): the oxygen molecule can be in either a singlet or a triplet state, and the NP may or may not contain an exciton. Optical pumping creates excitons, whilst PL emission and energy transfer processes annihilate them. Only energy transfer generates singlet oxygen, whilst spin relaxation (or infrared PL) processes return the oxygen to the triplet ground state. In the rate equation model for these processes, the photoexcited populations of the separate spin states of the excitons and the oxygen molecules are treated explicitly, taking into account the spin dependence of the energy transfer to O_2_, the radiative exciton recombination rate, the processes of thermal excitation and spin-lattice relaxation that lead to population redistribution between the spin states for a given silicon NP, and the rates of relaxation from singlet to triplet oxygen states.

### Silicon nanoparticles without oxygen

At the low measurement temperatures necessary for magneto-optical experiments (we use 1.5 K), we know that oxygen is not able to desorb from the nanoparticle surfaces (experimentally, we have to heat the sample to about 80 K before oxygen is released and can be pumped away, at which point the PL intensity recovers completely). We can therefore divide the NPs into two separate populations: those which are in contact with oxygen (represented in Figure 3) and those which are not. We write the proportion of NPs which do not have adsorbed oxygen molecules and which do not currently contain an exciton as *n*_0_; excitons are created in these in one of the three triplet exciton states (index *i* = 1…3) with equal pumping rates *P*/3 to generate fractional populations *u*_*i*_. The photoexcited NPs can de-populate only by radiative emission with rates *r*_0_,*r*_1_ for *m*_*j*_ = 0, *m*_*j*_ = ±1, respectively (note that, here, we set these equal; we will consider the consequences of these being different in a future work), spin-lattice relaxation to spin states lower in energy (*γ*_*ij*_), or thermal excitation to spin states higher in energy by Δ_*ij*_ (*γ*_*ij*_ = *γexp*(-Δ_*ij*_/*kT*)). Note that Δ_*ij*_ is dependent on the magnetic field since it arises from the Zeeman splitting of the exciton states; this leads to a magnetic field dependence of *γ*_*ij*_. Non-radiative relaxation processes may also contribute to the triplet exciton relaxation at low temperatures [11] but would enter into our model in the same way as the radiative decay rates and so are not included explicitly. Under these assumptions, the steady state solution of the rate equations for the fractional populations *u*_*i*_,*n*_0_ yields the following result (Equation 1):

(1) $$\begin{array}{l} (P/3)n_{0}-u_{1}(r_{1}+\gamma_{12}+\gamma_{13})+u_{2}\gamma_{21}+u_{3}\gamma_{31}=0\\ (P/3)n_{0}+u_{1}\gamma_{12}-u_{2}(r_{0}+\gamma_{21}+\gamma_{23})+u_{3}\gamma_{32}=0\\ (P/3)n_{0}+u_{1}\gamma_{13}+u_{2}\gamma_{23}-u_{3}(r_{1}+\gamma_{31}+\gamma_{32})=0\\ n_{0}+u_{1}+u_{2}+u_{3}=1-F, \end{array}$$

where *F* is the total fraction of NPs with adsorbed oxygen.

### Silicon nanoparticles with oxygen

**Figure 4:**
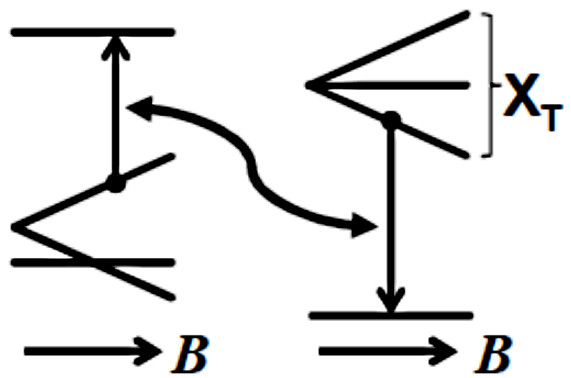
**Energy level diagram for the energy transfer from photoexcited silicon nanoparticles to oxygen molecules.** Left: the triplet (bottom) and singlet (top) levels of molecular oxygen in a magnetic field, showing the zero-field splitting between the *m*_*J*_ = 0 and the *m*_*J*_ = ±1 levels; right: the ground state (bottom) and triplet exciton (top) states of a silicon nanoparticle in a magnetic field. In this model, the exchange coupling between the two triplet states results in energy transfer between three possible pairs of states, of which one example is indicated by the vertical and curved arrows.

We now consider the second population of NPs, those which are in contact with oxygen. We write the proportions of NPs which do not contain an exciton as *n*_*j*_, where *j* runs over the three possible oxygen triplet states. As above, excitons are created in these NPs in one of the three triplet exciton states (index *i* = 1…3) with equal pumping rates *P*/3 to generate fractional coupled exciton-oxygen populations *n*_*ij*_. The exciton radiative recombination and spin-lattice relaxation terms are as above, and we introduce a spin-lattice relaxation and thermal excitation term between the oxygen triplet states analogous to *γ*_*ij*_ (*β*_*ij*_). Note, again, that *β*_*ij*_ is in general a function of magnetic field and depends on both zero-field and Zeeman terms (shown in Figure 4). We must also account for NPs in which the oxygen is in the singlet state and no exciton is present (the condition of an NP after energy transfer and before relaxation of the oxygen, with population *n*_*e*_) and NPs in which an exciton has been excited whilst the oxygen is still in the singlet state (populations *w*_*j*_).

Finally, we introduce the energy transfer process which is the focus of this work through the rate *t*_*ij*_. In the simplest approximation, as represented in Figure 4, the magnetic field and the principal axis of the oxygen molecule can be taken to be parallel; to model the behaviour with a random distribution of angles between these directions is substantially more complicated (requiring an average over the relative orientations and a calculation of the mixing of spin states) and will be discussed in future work. Here, our aim is to investigate what can be achieved with a realistic set of parameters in a comparatively simple model. The matrix *t*_*ij*_ here has the following form in order to impose the overall conservation of spin angular momentum, Δ*m*_*J*_ = 0:

(2) $$t_{\text{ij}}=\left(\begin{array}{ccc} 0 & 0 & t\\ 0 & t & 0\\ t & 0 & 0 \end{array}\right).$$

As in the previous subsection, we present the steady state solutions of the resulting 15 rate equations plus the condition that the total number of NPs with adsorbed oxygen remains constant. The first sets of expressions (Equations 3 to 5) represent the generation and loss of excitons in NPs with adsorbed triplet oxygen; the existence of two triplet entities gives nine possible joint spin states, so that nine equations are required.

(3) $$\begin{array}{ll} \;(P/3)n_{1} & -\left(r_{1}+t_{11}+\gamma_{12}+\gamma_{13}+\beta_{12}+\beta_{13}\right)n_{11}\\ & +\gamma_{21}n_{21}+\gamma_{31}n_{31}+\beta_{21}n_{12}+\beta_{31}n_{13}+(R/3)w_{1}=0\\ \;(P/3)n_{1} & -\left(r_{0}+t_{21}+\gamma_{21}+\gamma_{23}+\beta_{12}+\beta_{13}\right)n_{21}\\ & +\gamma_{12}n_{11}+\gamma_{32}n_{31}+\beta_{21}n_{22}+\beta_{31}n_{23}+(R/3)w_{2}=0\\ \;(P/3)n_{1} & -\left(r_{1}+t_{31}+\gamma_{31}+\gamma_{32}+\beta_{12}+\beta_{13}\right)n_{31}\\ & +\gamma_{13}n_{11}+\gamma_{23}n_{21}+\beta_{21}n_{32}+\beta_{31}n_{33}+(R/3)w_{3}=0 \end{array}$$

(4) $$\begin{array}{ll} \;(P/3)n_{2} & -\left(r_{1}+t_{12}+\gamma_{12}+\gamma_{13}+\beta_{21}+\beta_{23}\right)n_{12}\\ & +\gamma_{21}n_{22}+\gamma_{31}n_{32}+\beta_{12}n_{11}+\beta_{32}n_{13}+(R/3)w_{1}=0\\ \;(P/3)n_{2} & -\left(r_{0}+t_{22}+\gamma_{21}+\gamma_{23}+\beta_{21}+\beta_{23}\right)n_{22}\\ & +\gamma_{12}n_{12}+\gamma_{32}n_{32}+\beta_{12}n_{21}+\beta_{32}n_{23}+(R/3)w_{2}=0\\ \;(P/3)n_{2} & -\left(r_{1}+t_{32}+\gamma_{31}+\gamma_{32}+\beta_{21}+\beta_{23}\right)n_{32}\\ & +\gamma_{13}n_{12}+\gamma_{23}n_{22}+\beta_{12}n_{31}+\beta_{32}n_{33}+(R/3)w_{3}=0 \end{array}$$

(5) $$\begin{array}{ll} \;(P/3)n_{3} & -\left(r_{1}+t_{13}+\gamma_{12}+\gamma_{13}+\beta_{31}+\beta_{32}\right)n_{13}\\ & +\gamma_{21}n_{23}+\gamma_{31}n_{33}+\beta_{13}n_{11}+\beta_{23}n_{12}+(R/3)w_{1}=0\\ \;(P/3)n_{3} & -\left(r_{0}+t_{23}+\gamma_{21}+\gamma_{23}+\beta_{31}+\beta_{32}\right)n_{23}\\ & +\gamma_{12}n_{13}+\gamma_{32}n_{33}+\beta_{13}n_{21}+\beta_{23}n_{22}+(R/3)w_{2}=0\\ \;(P/3)n_{3} & -\left(r_{1}+t_{33}+\gamma_{31}+\gamma_{32}+\beta_{31}+\beta_{32}\right)n_{33}\\ & +\gamma_{13}n_{13}+\gamma_{23}n_{23}+\beta_{13}n_{31}+\beta_{23}n_{32}+(R/3)w_{3}=0 \end{array}$$

The next set of equations (Equation 6) represents the optical pumping and de-excitation of NPs with adsorbed oxygen in its singlet state; the three equations arise from the three exciton states.

(6) $$\begin{array}{l} (P/3)n_{e}-\left(r_{1}+R+\gamma_{13}+\gamma_{12}\right)w_{1}+\gamma_{21}w_{2}+\gamma_{31}w_{3}=0\\ (P/3)n_{e}+\gamma_{12}w_{1}-\left(r_{0}+R+\gamma_{23}+\gamma_{21}\right)w_{2}+\gamma_{32}w_{3}=0\\ (P/3)n_{e}+\gamma_{13}w_{1}+\gamma_{23}w_{2}-\left(r_{1}+R+\gamma_{31}+\gamma_{32}\right)w_{3}=0 \end{array}$$

The final set of equations represents the generation and loss of NPs with triplet oxygen but no exciton; the rate *R* expresses the oxygen relaxation from singlet to triplet state.

(7) $$\begin{array}{ll} \;(R/3)n_{e} & -\left(P+\beta_{12}+\beta_{13}\right)n_{1}+\beta_{21}n_{2}+\beta_{31}n_{3}\\ & +r_{1}n_{11}+r_{1}n_{31}+r_{0}n_{21}=0\\ \;(R/3)n_{e} & +\beta_{12}n_{1}-\left(P+\beta_{21}+\beta_{23}\right)n_{2}+\beta_{32}n_{3}\\ & +r_{1}n_{12}+r_{1}n_{32}+r_{0}n_{22}=0\\ \;(R/3)n_{e} & +\beta_{13}n_{1}+\beta_{23}n_{2}-\left(P+\beta_{31}+\beta_{32}\right)n_{3}\\ & +r_{1}n_{13}+r_{1}n_{33}+r_{0}n_{23}=0 \end{array}$$

As stated above, the remaining equation (Equation 8) imposes the requirement that the total fraction of NPs with adsorbed oxygen should remain constant at *F*. With this condition, we have a fully determined system and can solve for all 16 variables in this equation.

(8) $$\begin{array}{ll} \;n_{e} & +n_{1}+n_{2}+n_{3}+w_{1}+w_{2}+w_{3}\\ & +n_{11}+n_{12}+n_{13}+n_{21}+n_{22}\\ & +n_{23}+n_{31}+n_{32}+n_{33}=F \end{array}$$

We can sum all the exciton radiative processes in order to obtain an expression for the PL intensity *I*_PL_ as follows:

(9) $$\begin{array}{ll} \;I_{\text{PL}} & =r_{1}\left(n_{13}+n_{33}\right)+r_{0}n_{23}\\ & \;+r_{1}\left(n_{12}+n_{32}\right)+r_{0}n_{22}\\ & \;+r_{1}\left(n_{11}+n_{31}\right)+r_{0}n_{21}\\ & \;+r_{1}\left(w_{1}+w_{3}\right)+r_{0}w_{2}\\ & \;+r_{1}\left(u_{1}+u_{3}\right)+r_{0}u_{2} \end{array}$$

and this expression can be evaluated as a function of magnetic field; note that *n*_*ij*_, *w*_*i*_ and, in principle, *u*_*i*_ are all functions of magnetic field through the field dependence of *γ*_*ij*_ and *β*_*ij*_.

### Comparison to experiment

**Figure 5:**
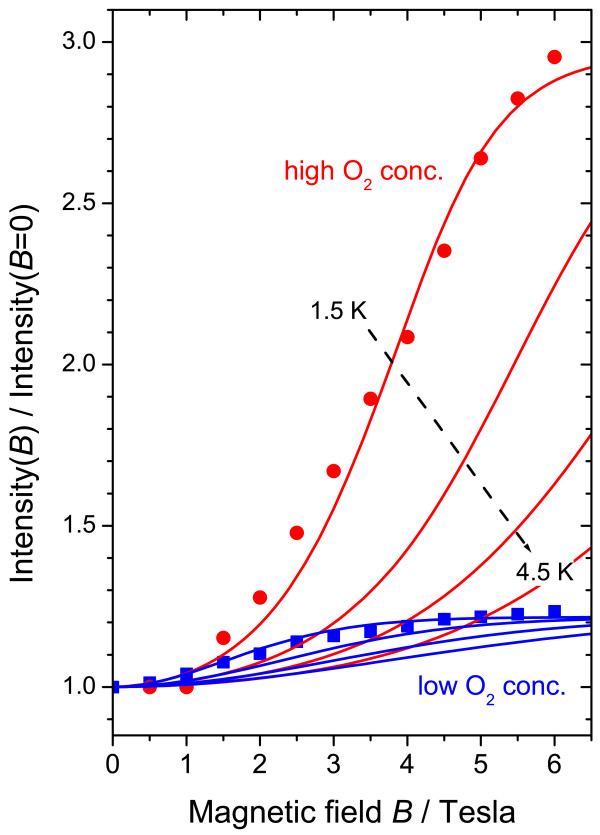
**Comparison of experimental data and results of the rate equation model.** Solid points: the ratio of the PL intensity at magnetic field *I*(*B*) to that at zero field *I*(*B* = 0) (red circles and blue squares: high and low O_2_ concentrations, respectively); lines: predictions of the rate equation model for *I*(*B*)/*I*(*B* = 0) keeping all parameters constant except those related to the oxygen concentration and for a series of temperatures (upper to lower curves) of 1.5 to 4.5 K in 1-K steps.

The above model does not account for phonon-assisted processes and therefore is strictly only valid for NPs emitting PL at the threshold energy of 1.63 eV. In fact, this is not a serious limitation, since the degree of recovery of the PL in a magnetic field is similar over a PL energy range wide in comparison to a phonon energy. It is beyond the scope of this work to discuss the energy dependence of the transfer process in detail, and so we extract only the PL intensities at 1.63 eV from the spectra of Figures 1 and 2 and plot them in Figure 5 as a function of magnetic field, normalized to the PL intensity at zero field. This normalization eliminates the difficulties associated with considering absolute PL intensities and will facilitate the comparison of data from different samples.

Figure 5 also shows calculated results based on the above model, in which we take a set of parameters based on the recent literature. These are summarised in Table 1. For the two sets of experimental data, we maintain all parameters at the same values, except for those associated with the energy transfer process itself: these are *F*, which expresses the proportion of NPs without oxygen, and the transfer rate *t*, which decreases as the probability of an NP having multiple O_2_ molecules available increases.

**Table 1:** Parameters used in modelling (inverse rates, in seconds)

	**This work**		**Typical**	**Source**
	**Low O**_**2**_	**High O**_**2**_		
Silicon NP				
$r_{1}^{-1}$	10^-5^	10^-5^	10^-5^ to 10^-2^	[13]
$r_{0}^{-1}$	10^-5^	10^-5^		
*γ*^-1^	10^-7^	10^-7^		
*P*^-1^	1/45	1/45		
Oxygen				
*F*	0.75	0.85		
*R*^-1^	4 × 10^-3^	4 × 10^-3^		
*β*^-1^	2 × 10^-7^	2 × 10^-7^		
*t*^-1^	10^-5^	2 × 10^-7^	2.6 × 10^-6^	[12]

The fraction *F* of NPs with adsorbed oxygen was varied from 0.75 (Figures 1 and 5, blue) to 0.85 (Figures 2 and 5, red), and 1/*t* varied from 10^-5^ to 10^-7^ s. More work is needed before we would attempt to interpret these parameters directly, but we note that these transfer times are in good agreement with previously measured values [12], and as is necessary for the evenly matched competition between radiative recombination and energy transfer, they are comparable to the radiative lifetimes 1/*r*_1_,1/*r*_0_[13]. In the simulations, we also varied the temperature, since the field at which the PL recovery approaches saturation is sensitive to the relationship between *gμ*_*B*_*B* and *kT*. As can be seen from Figure 5, the simulations agree well with the experimental results taking the nominal experimental temperature of 1.5 K. We will report elsewhere on studies of the excitation intensity dependence of the effect; there, we find we must take into account an increase in temperature for high excitation intensities (here, these were the same for Figures 1 and 2 and were low).

## Conclusions

Using the simple model set out above, the dependence of the photoluminescence spectra of silicon nanoparticles with adsorbed oxygen molecules has been studied and it is shown that a realistic set of parameters can give an adequate description of the recovery of the PL intensity with increasing magnetic field, confirming the proposed spin-dependent exchange-coupled mechanism for the energy transfer process. In particular, one set of parameters can describe the behaviour of the magnetic field dependence for high and low oxygen coverage of the sample by changing only the parameters directly relevant to the energy transfer process. This represents the first detailed and quantitative investigation of magnetic field effects in the photogeneration of singlet oxygen by use of silicon nanoparticles and provides a model which can easily be expanded in order to investigate the dependence of the energy transfer process on nanoparticle size, excitation intensity, and temperature; this work is in progress.

## Competing interests

The authors declare that they have no competing interests.

## Authors’ contributions

JA, GNA, and DW carried out the magneto-luminescence measurements. JA, GNA, and PAS prepared the porous Si samples, and JJD, DW, GNA, and JA all contributed to development and testing of the model. All authors contributed to planning this work and read and approved the final manuscript.
